# Treatment of Aseptic Femur Non-union With Broken Intramedullary Nail In Situ by Exchange Nailing: A Technical Description

**DOI:** 10.7759/cureus.91011

**Published:** 2025-08-26

**Authors:** Salman Durrani, Jitender Saini, Nischay Kaushik, Juvesh Kumar, Pramod Kumar, Shubham Agrawal

**Affiliations:** 1 Orthopedic Surgery, Dr Baba Saheb Ambedkar Medical College and Hospital, New Delhi, IND; 2 Orthopedics and Trauma, Dr Baba Saheb Ambedkar Medical College and Hospital, New Delhi, IND; 3 Orthopedic Surgery, Dr Baba Saheb Ambedkar Hospital and Medical College, New Delhi, IND; 4 Orthopedics, Jawaharlal Nehru Medical College, Aligarh Muslim University, Aligarh, IND; 5 Orthopedics, Dr Baba Saheb Ambedkar Medical College and Hospital, New Delhi, IND

**Keywords:** antegrade intramedullary nail, broken implant, implant removal, intramedullary femoral nail, non-union shaft femur

## Abstract

Non-union following intramedullary nailing of diaphyseal long bone fractures represents a serious complication necessitating timely and effective intervention. The situation becomes considerably more complex in the presence of a broken femoral nail, which poses additional technical challenges for the operating surgeon. Conventional management strategies typically entail removal of the existing nail, followed by re-fixation with a larger intramedullary nail, plate fixation, or external fixation constructs.

Our case describes a 34-year-old male patient who presented with aseptic femur non-union complicated by refracture and hardware failure. The intramedullary nail had broken at two distinct sites as revealed by radiographs. A novel technique was employed to remove the broken hardware without surgically exposing the fracture site, followed by exchange nailing with a larger-diameter implant. A distal entry point in the femur was created in addition to the proximal entry site. After removal of the broken locking screws, a jig was attached proximally, and a solid reamer was introduced distally to engage the tip of the retained nail fragment. Simultaneous hammering was performed from both proximal and distal directions, enabling the extraction of all three nail fragments through the proximal entry point. The procedure was completed with the insertion of a wider intramedullary nail. The patient underwent successful fixation with exchange nailing, demonstrated progressive radiological healing, and ultimately achieved fracture union. At final follow-up, he had returned to full function without pain or restriction of daily activities.

The described approach not only overcomes the challenges of implant removal but also raises awareness and shows a method to overcome its difficulty. This particular technique also reduces the risk of intraoperative and postoperative complications.

## Introduction

Modern intramedullary nailing techniques for femoral shaft fractures have significantly reduced the incidence of non-union, with rates reported as low as 1% [1]. However, more recent studies suggest that the incidence is increased, with some reports indicating rates as high as 25% [2]. This increase is thought to be due to better survival rates among severely polytraumatized patients and the growing preference for early internal fixation [3].

Several treatment methods have been described for femoral shaft non-union, including the use of external fixators and plate fixation [4-7]. The most commonly employed approach, especially for aseptic non-union, involves the insertion of a reamed, locked intramedullary nail, often combined with open autologous bone grafting [2].

Locked intramedullary nailing is widely regarded as the preferred treatment for most femoral shaft fractures [8]. It is favored over plating due to the latter’s higher risks of infection and non-union [9]. Factors that increase the risk of non-union following intramedullary nailing include open fractures, distal-third femoral fractures treated with antegrade nailing, delayed initiation of weight-bearing, and tobacco use [10]. Management options for non-union with a statically locked intramedullary nail in place include in situ bone grafting, dynamization by removal of locking screws, or exchange nailing.

This study was undertaken due to the limited data available in the literature on aseptic broken femoral nail removal. Even in the few reports that exist, the specific technique we describe has not been detailed. By presenting our method, we aim to raise awareness among readers about an innovative approach to implant removal that may serve as a valuable option in such challenging scenarios. An additional advantage of this technique is that it avoids opening the fracture site, thereby reducing morbidity for the patient.

## Case presentation

Preoperative course

A 34-year-old male patient presented to the department of Orthopedics, Dr Baba Saheb Ambedkar Medical College and Hospital, during October 2024, with complaints of pain and inability to bear weight in the right lower limb, after sustaining a reported ground-level fall two days ago. The patient's history was significant for a femur shaft fracture five months ago, which was managed with closed reduction and internal fixation with an intramedullary nail. He reported intermittent pain mainly located in the mid-thigh and reported little difficulty in walking before falling again.

**Figure 1:**
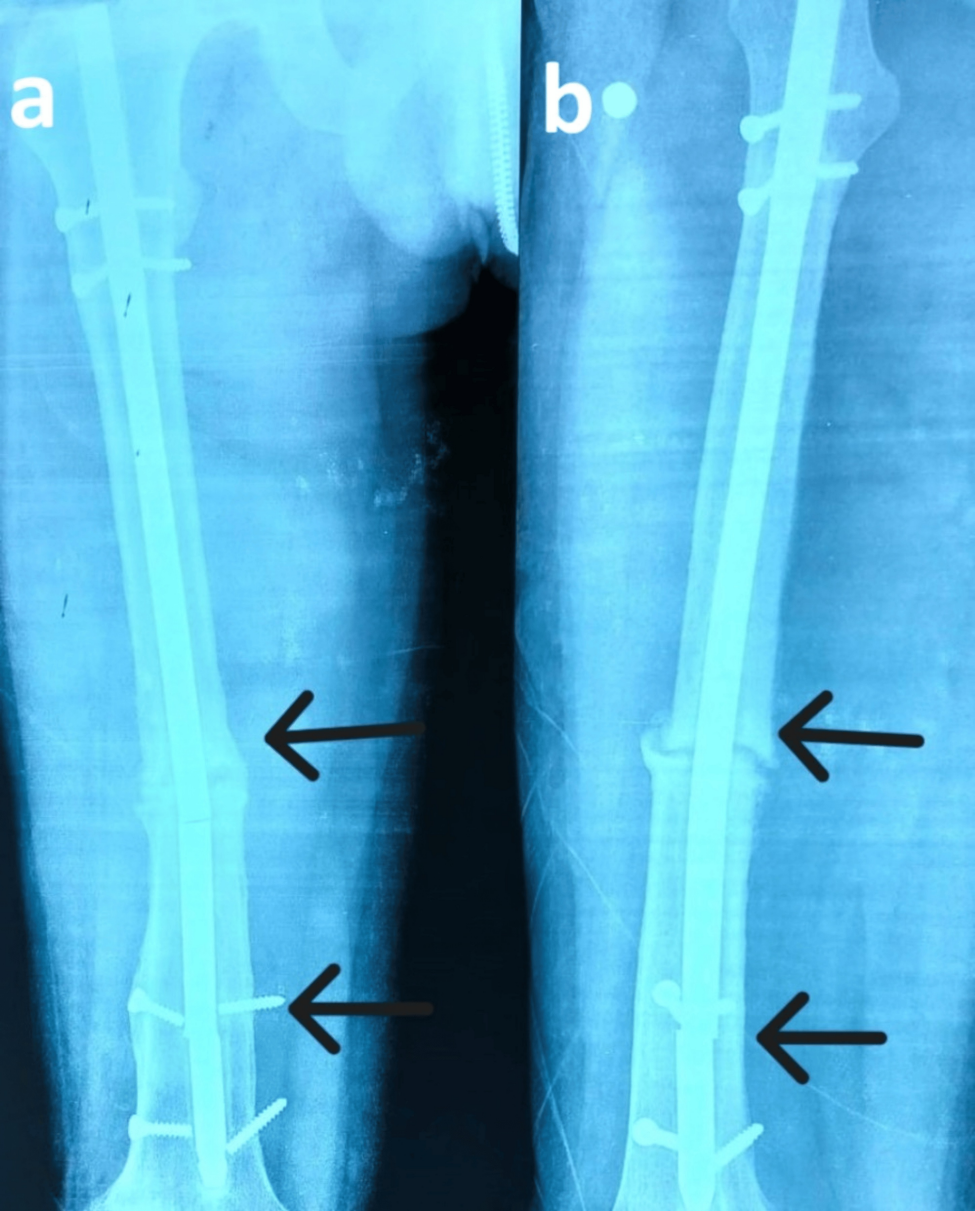
Preoperative image showing aseptic non-union with implant broken at two sites The X-ray shows preoperative non-union of the shaft of the femur with the implant broken at two sites, as marked by arrows at two different places, with arrows in AP view (a) and lateral view (b). AP: Anteroposterior

On examination, there was swelling in the mid-thigh, deformity, and shortening of the right lower limb. A healed scar from previous surgery was present, and tenderness was noted on palpation. Passive and active joint motion was painful and restricted. An X-ray was performed, which showed non-union with breakage of nail and all the locking screws, as shown in Figure 1.

After written and informed consent as well as clearance from anesthesia, the patient was planned for surgery.

Intraoperative course

We strategized that an intramedullary nail with a larger size was the best course of action. The tibia skeletal traction was removed before the procedure, and the patient was placed supine on a standard radiolucent table and spinal anesthesia was given. First, the entry point was identified. Excess bone was removed with bone nibbler. The femoral zig was inserted and secured at the nail tip in order to facilitate nail removal. Then proximal and distal broken bolt sites were identified with image intensifier, and the proximal parts of the bolts were removed. we needed to nibble out some bone in the process.

**Figure 2:**
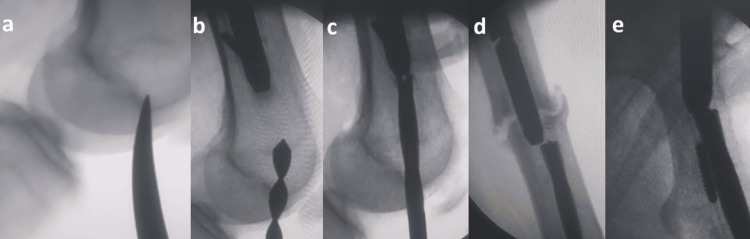
Intraoperative images demonstrating the technique for removal of the broken nail a: Initial distal femoral entry created using an awl; b: The canal opened with a canal opener; c-e: Sequential use of a reamer with hammering allowed the retained nail fragment to be dislodged proximally and removed proximally through the antegrade entry site.

After that, we had to remove the distal and middle part of the nail, which was broken. We removed it in antegrade fashion. However, we opted to remove without opening the fracture site by establishing an entry point in the distal part of the femur and removing it antegrade with the proximal part of the nail. We first made the distal entry point in the femur as we do for the distal femur nail with an awl. A canal opener was first inserted, followed by 8 mm and then 9 mm reamers. The reamer was then sequentially hammered and back-hammered at the proximal part of the nail in an attempt to extract both the distal and middle fragments together with the proximal portion from the proximal side. After some time, the distal, middle, and proximal broken parts of the nail began to move upwards, and finally, all three parts of the nail were removed using a sequential hammer and backhammer as shown in Figure 2.

**Figure 3:**
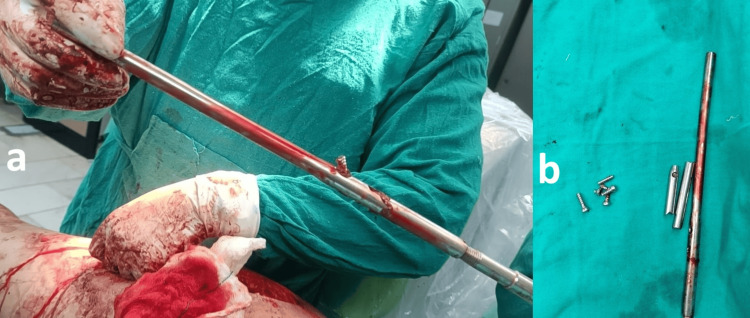
Intraoperative images showing removal of nail in antegrade site and the nail in three parts with broken screws a: Removal of the nail from the proximal site; b: Removed nail in three broken parts

All three parts of the nail were removed with broken parts of screw as shown in Figure 3.

**Figure 4:**
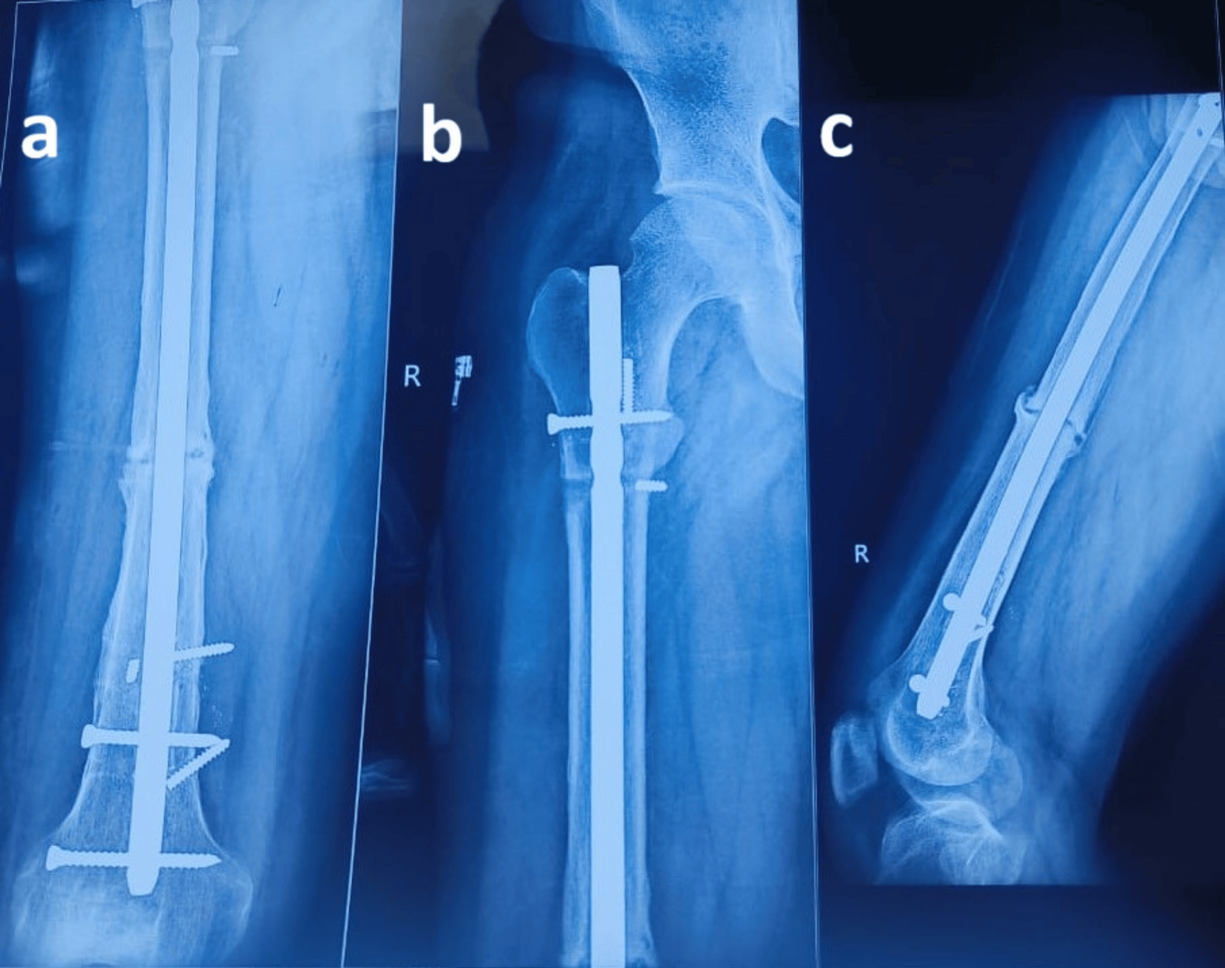
Postoperative images showing exchange nailing done with large-size nail and well-fixed implant AP views (a, b) demonstrate insertion of a larger diameter intramedullary nail, occupying the distal two-thirds and proximal one-third of the femur, with satisfactory alignment. The lateral view (c) shows a well-fixed 10 × 380 mm nail, inserted after removal of the previous 9 × 380 mm nail. The use of a larger nail provided improved stability and enhanced fixation strength at the fracture site. AP: Anteroposterior

We reamed with a bigger reamer sequentially, with 8, 9, 10, and finally 11 mm reamer, and then we inserted an size 10x38-mm nail. Post that, distal and proximal locking of bolts was done, as shown in Figure 4.

Wound closure was done in layers, and an antiseptic dressing was done. The patient was shifted out of the operating theater in a stable condition.

Postoperative course

On the first postoperative day, the patient was advised to sit up; passive range of motion (ROM) was also started. On the second postoperative day, in-bed turning; active quadriceps, hamstrings, and ankle pump; and full weight bearing were initiated, and on the fifth postoperative day, he was discharged after a second dressing. 

## Discussion

Managing femoral non-union following initial intramedullary fixation is a difficult task for orthopedic surgeons, and the presence of a broken implant in situ further complicates the situation. Retrieval of broken distal fragment in the shaft of femur fracture, non-union or united fractures is also a challenging task. In the present study, the removal of the proximal fragment was done by inserting the nail zig in the proximal part and back hammering it after the removal of the proximal screws. However, Sivananthan et al. and Levy et al. used smaller diameter nails [11,12]. The locking bolts of the distal broken part were removed, and with the help of smaller diameter nails, impaction was done into the distal broken nail for removal. Charnley and Farrington utilized laparoscopic forceps for a similar extraction purpose [13]. Franklin et al. employed a hooked wire passed through the cannulated nail to engage and extract its tip [14]. Jain et al. described using a stainless-steel wire bent at the tip into a fishhook shape to remove the broken distal fragment of an intramedullary nail [15]. Marwan and Ibrahim reported using two stainless steel wires-one looped and inserted externally through a locking bolt hole, and the other passed through the nail itself [16]. The internal wire was retrieved using the looped wire, and both were twisted together to assist in extraction. In addition to these techniques, various other tools have been used, such as Enders' nails, multiple olive wires, sigmoidoscopy forceps, and smaller-sized flexible or cannulated reamers [17]. Magu et al. described a method involving knee arthrotomy and drilling through the distal femur to retrogradely pass an olive-tipped wire for nail removal [18].

Earlier studies, such as Pongsamakthai et al., have endorsed the use of Kuntscher nails, narrow-diameter nails, small reamers, and extraction hooks in situations involving small nail diameter [19]. However, the conventional method of using a hook to retrieve broken implants is often ineffective due to several limitations, including insufficient grip, difficulty in engaging a nail tip that is deeply embedded in the subchondral bone, and the inability to extract a large distal fragment due to inadequate strength. One should also be extra careful while removing Kuntscher nail as distal part of nail becomes wide and very difficult to remove [20,21]. In our case, we were fortunate that the distal part of the nail was hammered with a solid reamer from the distal entry side and removed from the proximal part.

Preoperative planning should consider several critical details, including the nail design (solid or cannulated), nail diameter, the location of the broken implant, and the status of fracture healing. These factors significantly influence the selection of the most appropriate removal technique.

A proximally-fitted extraction method can be employed, which involves engaging the proximal end of the retained distal nail fragment using instruments such as a T-reamer, an elastic nail, or a smaller-sized nail. One distal locking screw should be retained to provide counter-resistance while the instrument is impacted to engage the proximal end of the broken nail fragment. Once the instrument is firmly jammed into the fragment, a back-slapping technique is used to extract the distal piece in a retrograde manner.

Additionally, reaming the proximal canal with a reamer two sizes larger than the original nail has been recommended to facilitate the removal of the distal fragment by enlarging the canal diameter [11].

Based on our study, we recommend considering removal of the full nail in the proximal part by making an entry in the distal part and with the help of a solid hammer and reamer as well as antegrade removal of the nail without opening the fracture site during preoperative planning for the removal of bent or broken intramedullary nails to avoid unexpected complications.

## Conclusions

The removal of a broken intramedullary nail is often technically demanding. In this report, we described a step-by-step technique for extracting the retained nail fragment without surgically exposing the fracture site. Using a distal entry point, a solid reamer was introduced and, with the assistance of sequential hammering, traction was applied proximally via the attached jig and distally via the reamer. By simultaneously hammering from both ends, the nail was successfully removed through the piriformis entry site. This technique highlights a practical approach that may facilitate easier removal of broken nails while minimizing patient morbidity.
